# The effects of the elastin polymorphisms on carotid intima-media thickness in women aged 30 – 70

**DOI:** 10.20463/jenb.2018.0012

**Published:** 2018-06-30

**Authors:** Seung-Taek Lim, Jin-Kee Park, Sang-Hyuk Park, Eun-Jae Lee, Woo-Nam Kim, Seok-Ki Min

**Affiliations:** 1 Institute for bio-health integration of medicine and Korean medicine, Nasaret international hospital, Incheon Republic of Korea; 2 Department of sports rehabilitation, Dong-Ju College, Busan Republic of Korea; 3 Department of sport science, Korea Institute of Sport Science (KISS), Seoul Republic of Korea; 4 Center for sport science in Incheon, Incheon sports council, Incheon Republic of Korea; 5 College of sport science, Dong-A University, Busan Republic of Korea

**Keywords:** carotid intima-media thickness, elastin, polymorphism, vascular features, women

## Abstract

**[Purpose]:**

Elastin is one of the major determinants of arterial distensibility of large blood vessels that forms the principal component of elastic fibers from the media of arteries. However, the association between elastin(ELN) genotype and vascular function is still unclear.

**[Methods]:**

120women were recruited from the Saha-gu (Busan, Korea) Community Center. Measurements of body composition and vascular function included carotid intima-media thickness (CIMT), carotid artery luminal diameter (CLD), minimum (diastolic) artery luminal diameter (CLD_min_) and maximum (systolic) artery luminal diameter (CLD_max_). Genotyping for the ELN (rs 2071307) polymorphism was performed using the TaqMan approach. ELN gene distribution of subjects were in the Hardy-Weinberg equilibrium (p=0.402).

**[Results]:**

The relative CIMT differed significantly among the ELN genotypes. And not significant differences in CLD and CIMT/CLD ratio, but AA genotype was tended higher than other genotypes (AG and GG). The relative CIMT and CLD min differed significantly between the ELN alleles. And not significant differences in CLD max and CIMT/CLD ratio, but A allele was tended higher than G allele.

**[Conclusion]:**

These results suggest that ELN gene polymorphism might be used a one of the genetic determinants of vascular disease in both pre- and postmenopausal women.

## INTRODUCTION

Currently, the various environmental factors of vascular disease(i.e., atherosclerosis, hypertension, stroke, and pulmonary arterial hypertension) are implicated in the development of its pathology; furthermore, a strong genetic diathesis based on family history has been identified^1^. One of the animal studies suggested that the genetic factors might influence the effects from the age increase and hypertension^2,3^.

Elastin is a molecular determinant of arterial morphogenesis that stabilizes the arterial structure by regulating the vascular proliferation and organization^4^. The elastin genotype (ELN, rs 2071307) has been located on the chromosome 7 in humans^5^, and Tromp et al^6^. identified the A-to-G polymorphism of the elastin gene in the exon 16, which is the result of a variant that converts the codon-AGT-(serine) at the aminoacid position 422 to the -GGT-(glycine). Regarding the ELN gene, it has been reported that, as the artery stiffens, the hemodynamic load that its endothelium is exposed to increases, thereby damaging the endothelium; furthermore, this can become a vicious cycle^7^.

The reports show that the ELN genotype is associated with not only the vascular function but also the vascular structure. Hanon et al.^2^ studied a cohort of 320 subjects who did not show any evidence of cardiovascular disease (CVD), thereby producing the following results: 10 % for the AA genotype, 51 % for the AG genotype, and 39 % for the GG genotype. Also, the carotid-artery distensibility of the Hanon et al.^2^ cohort is significantly decreased, and the elastic modulus is significantly increased in the A-allele subjects compared with the G-allele subjects. Deng et al.^8^ study of 358 subjects with isolated systolic hypertension shows 2.8 % for the AA genotype, 15.1 % for the AG genotype, and 82.1 % for the GG genotype. It has been suggested that these results are due to the reported association between the ELN genotype and arterial stiffness. A genetic factor based difference regarding the vascular structure is not evident in the CVD patients, because CVD itself might be a risk factor for the negative exchange of the arterial structure.

Previous studies reported the association of the ELN genotype with the vascular function. The carotid-artery intima-media thickness (CIMT) and the carotid-artery luminal diameter (CLD), however, are markers of subclinical atherosclerosis, and are also powerful predictors of future CVD events^9^. The estimates of the CVD heritability range from 30–86 %^10^. Numerous studies have been conducted on the effects of a range of candidate genes (i.e. APOE: apolipoprotein E; ACE: angiotensin I convertingenzyme; MTHFR: 5,10-methylenetetrahydrofolate reductase; NOS3: nitric oxide synthase 3 [endothelialcell]; ADD1: adducin 1) on the CIMT, the results of which could provide useful insights into the genetic influences regarding atherothrombotic disease, as well as large artery ischemic stroke and ischemic heart disease. Thus, the carotid-artery structure is a stronger predictor for atherosclerosis in healthy people.

The association between the ELN genotype and the vascular structure in the general female population, however, remains unclear. Therefore, the aim of the present study is the investigation of the association between the ELN genotype and the CIMT according to measurements in women aged from 30-70 years.

## METHODS

### Subjects

120women were recruited from the Saha-gu (Busan, Korea) Community Center (Table 1). For subjects were following exclusion criteria: (1) history of stroke, cardiovascular diseases, or hepatectomy; (2) history of hypertension; (3) taking medication. All subjects who agreed to participate in the study had the study explained to them to ensure a complete understanding of its purpose and the methods used. The subjects also signed an informed consent form before participation. The study received approval from the Korea Institute of Sport Science (IRB KISS- 201601-IFS-002-P1) before participant recruitment began.

**Table 1.** The characteristic of the subjects.

Variable (*n*=120)	
Age (years)	54.6 ± 8.6
Height (cm)	157.7 ± 5.8
Weight (kg)	59.0 ± 8.5
BMI (kg/m^2^)	23.6 ± 2.8
%BF (%)	32.0 ± 5.4
BMM (kg)	21.7 ± 3.7
SBP (mmHg)	128.5 ± 11.7
DBP (mmHg)	79.2 ± 8.9

Values are mean ± SD. BMI, body mass index; %BF, percent body fat; BMM, body muscle mass; SBP, systolic blood pressure; DBP, diastolic blood pressure

### Measurements of body composition

Body weight and height were measured to the nearest 0.1 kg and 0.1 cm, respectively, using an Inbody 370 body composition analyzer (Inbody Co. Ltd, Seoul, Korea). BMI was calculated as weight (kg) divided by height squared (m^2^).

### Measurements of vascular function

CIMT was measured using B-mode ultrasound and a10-MHz probe (LOGIQ 3, GE Healthcare, Wauwatosa, WI, USA). During measurement of the carotid arteries, the subjects lay on their backs in a dark room, turned their heads 45 degrees, and fully exposed the carotid arteries after they relaxed for minimum 10 minutes; left carotid artery was then measured by ultrasound^12^. Three IMT measurements of diastolic images on each side at 10 mm before or after the carotid bifurcation were obtained. The mean IMT was calculated for each point, and the highest value (maximum IMT) was recorded for each subject and defined as the distance from the lumen-intima interface to the in tima adventitia interface. The carotid artery lumen diameter (CLD) at diastole was measured by M-mode ultrasound. The lumen diameter between the near and far wall intima-media interfaces was imaged along the vessel length, the 10 mm segment proximal to the dilation of the carotid bulb on both sides. Measurements included minimum (diastolic) artery luminal diameter (CLD_min_) and maximum (systolic) artery luminal diameter (CLD_max_).

### DNA extraction and ELN genotyping

Genomic DNA was extracted from buccal cells which were obtained by using cotton swabs (Single Warpped, COPAN, CA, USA). After cell preparation, the sample were dissolved, the cells were lysed in 400μl of DNA lysis solution and incubated at 95°C for 3 minutes. The samples were added 400μl of DNA stabilizing solution then stored at 4°C until polymerase chain reaction (PCR). Genotyping for the ELN polymorphism was performed by real-time PCR using a TaqMan probe (rs 2071307, Pre-Designed SNP Genotyping assays). The PCR cycling reaction, using thermal cycler (7500, Applied Biosystem, CA, USA) were as follows: 95°C for 10 min, 40 cycles at 95°C for 15s, at 60°C for 1 min.

### Statistical analysis

The SPSS statistical package version 19.0 for Windows (SPSS, Inc., Chicago, IL, USA) was used to perform all statistical evaluations. Allele frequencies were determined by gene counting. X^2^ test were used to confirm that the observed genotype frequencies exhibited a Hardy-Weinberg equilibrium distribution. For body composition and vascular features, data were further analyzed for significant difference between the three genotypes using a One-Way ANOVA and where appropriate a post hoc Bonferroni test. Statistical significance was accepted at the 0.05 level. All variables are presented as the means ± standard deviations.

## RESULTS

### ELN polymorphism

The distribution of the ELN polymorphism and allele are presented in Table 2. ELN gene distribution of subjects were in the Hardy-Weinberg equilibrium (*p*=0.402).

**Table 2.** Distribution of ELN genotypes in all subjects

	Genotype frequency, % (n)			Allele frequency, % (n)	
	AA	AG	GG	A	G
Subjects (n=120)	4.2(5)	26.6(32)	69.2(83)	17.5(42)	82.5(198)

ELN gene distribution of subjects were in Hardy-Weinberg equilibrium (*p*=0.402)

Allele, A = (2AA) + AG; G = (2GG) + AG

### Relationship of ELN polymorphism and body composition

The body composition of the subjects according to each genotypes are shown in Table 3. There was no significant difference among the groups with respect to genotypes.

**Table 3.** The comparison of body composition according to ELN genotypes

	ELN polymorphism			*p*
	AA (n=5)	AG (n=32)	GG (n=83)	
Age (years)	58.2 ± 13.9	55.9 ± 8.0	53.9 ± 8.5	0.326
Height (cm)	159.7 ± 8.8	156.9 ± 6.5	157.9 ± 5.3	0.543
Weight (kg)	63.6 ± 9.6	58.8 ± 7.9	58.9 ± 8.6	0.476
BMI (kg/m^2^)	22.8 ± 5.2	23.9 ± 2.6	23.6 ± 2.8	0.704
%BF (%)	31.5 ± 9.4	33.1 ± 5.8	31.6 ± 5.0	0.434
BMM (kg)	24.0 ± 7.1	21.3 ± 4.0	21.8 ± 3.3	0.291
SBP (mmHg)	125.5 ± 7.8	127.8 ± 13.2	128.9 ± 11.4	0.887
DBP (mmHg)	81.5 ± 9.2	77.7 ± 8.5	79.7 ± 9.2	0.690

Values are mean ± SD. BMI, body mass index; %BF, percent body fat; BMM, body muscle mass; SBP, systolic blood pressure; DBP, diastolic blood pressure

p - value was analyzed by One-way ANOVA

### Relationship of ELN polymorphism and vascular features

The vascular features of the subjects according to each genotypes are shown in Table 4a. The relative CIMT (*p*=0.039) differed significantly among the ELN genotypes. And not significant differences in CLD and CIMT/CLD ratio, but AA genotype was tended higher than other genotypes (AG and GG).

The vascular features of the subjects according to each allele are shown in Table 4b. The relative CIMT (*p*=0.010) and CLD_min_ (*p*=0.032) differed significantly between the ELN alleles. And not significant differences in CLD_max_ and CIMT/CLD ratio, but A allele was tended higher than G allele.

**Table 4.** The comparison of vascular features according to ELN genotypes

a. According to each genotypes				
	ELN polymorphism			p
	AA (n=5)	AG (n=32)	GG (n=83)	
CIMT (mm)	0.88± 0.38	0.77 ± 0.15	0.71± 0.15	0.039
CLD_min_ (cm)	0.64 ± 0.08	0.60 ± 0.06	0.58 ± 0.06	0.070
CLD_max_ (cm)	0.61 ± 0.05	0.56 ± 0.06	0.55 ± 0.06	0.092
CIMT/CLD_min_ ratio	1.20 ± 0.12	1.27 ± 0.35	1.24 ± 0.26	0.865
CIMT/CLD_max_ ratio	1.28 ± 0.18	1.33 ± 0.38	1.31 ± 0.28	0.930

Values are mean ± SD. CIMT, carotid artery intima-media thickness; CLD_min_, minimum (diastolic) artery luminal diameter; CLD_max_, maximum (systolic) artery luminal diameter;

p - value was analyzed by One-way ANOVA

**Table:** 

b. According to each allele				
	ELN polymorphism		p
	A allele (n=42)	G allele (n=198)	
CIMT (mm)	0.79 ± 0.22	0.72 ± 0.15	0.039
CLD_min_ (cm)	0.61 ± 0.06	0.59 ± 0.06	0.070
CLD_max_ (cm)	0.57 ± 0.06	0.56 ± 0.06	0.092
CIMT/CLD_min_ ratio	1.30 ± 0.29	1.24 ± 0.28	0.865
CIMT/CLD_max_ ratio	1.38 ± 0.31	1.31 ± 0.29	0.930

Values are mean ± SD. CIMT, carotid artery intima-media thickness; CLD_min_, minimum (diastolic) artery luminal diameter; CLD_max_, maximum (systolic) artery luminal diameter;

p - value was analyzed by One-way ANOVA

Allele, A = (2AA) + AG; G = (2GG) + AG

## DISCUSSION

The present study is the first of its type to investigate the association between the ELN genotype and the vascular features in a general female. The main finding of this study is an association between the CIMT and the ELN genotypes. Moreover, increasing trends regarding the CLD and the CIMT/CLD ratio are evident among the subjects with the ELN AA genotype, but these are not significant.

The CIMT of the common carotid artery is prominent as an early indicator of atherosclerosis and cardiovascular risk, and the CIMT reflects the different stages and aspects of atherosclerosis with distinct determinants^13^. Actually, the CIMT and atherosclerosis share common underlying mechanisms for both the disease initiation and progression^14^. It is known that the CLD is influenced by the CIMT as well as the metabolic and hemodynamic parameters^15^. Jensen-Urstad et al.^16^ showed a correlation between the CIMT and the CLD. Univariate Cox-regression hazard analyses revealed that the CIMT (hazard ratio: 13.91; 95% confidence interval: 1.43 to 135.43; *p*=0.023), CLD_min_ (hazard ratio: 2.05; 95% confidence interval: 1.41 to 2.96; *p*=0.0002) and CLD_max_ (hazard ratio: 1.90; 95% confidence interval: 1.35 to 2.69; *p*=0.0002)are most likely to lead to the development of major cardiovascular events^17^.

The carotid artery is an elastic artery with high amounts of elastin and collagen fibers, while the radial artery is a muscular artery that is principally composed of arterial smooth muscle^18^. Previous reports indicate an association between the ELN genotype and the aorta in humans^1^. Further, the Ser422Gly polymorphism (rs2071307) is associated with a decreased distensibility and an increased elastic modulus at the elastic artery sites^2^. Also, Deng et al.^8^ reported that the ELN gene is associated with an increased risk of the isolated systolic hypertension and aortic stiffness in the Chinese Han population. The present study shows that the CIMT differed significantly among the various ELN genotypes, and this is similar to the results that were demonstrated by Deng et al.^8^ and Hanon et al.^2^. Moreover, the authors found the heterozygous mutation in the GG genotypes.

Several studies have reported that the cancer associated SAMHD1 mutations increase the mutation rates in cancer cells^19^, and the heterozygous RTEL1 gene mutations have produced the same effect in a patient with the Hoyeraal- Hreidarsson Syndrome^20^.The other vascular-function studies do not show any significant differences among the ELN genotypes, but the AA-genotype tendency regarding the peak-systolic velocity (PSV) is higher than those of the other genotypes (AG and GG).The CIMT is a strong coronary-artery disease (CAD) predictor in both pre- and post menopausal women, which is in contrast to the menopausal status^21^. In addition, the Atherosclerosis Risk in Communities (ARIC) Study demonstrated that the mean CIMT of ≥ 1.0 mm is associated with a cardiac event hazard ratio that is over-2.5-fold higher in women (5.07) compared with men (1.85)^22^. This might be a particularly important finding regarding women.

Aging is another main cause of the functional changes in the arterial wall that lead to an increased arterial stiffness^23^. In addition, the elastic modulus of the carotid artery is significantly higher in those subjects carrying the A allele compared with the subjects who were > 50 years of age^2^. The present results indicate that the mutations affecting the overall architecture of the elastic network could be involved in the CIMT caused vascular change in the arterial wall that is higher in the AA-genotype subjects. In particular, the increased CIMT seriously affected the health and lifestyle of a significant part of the female population, especially after menopause^24^. Several studies have reported that the low common carotid end-diastolic velocity (EDV) and PSV are independently associated with the probability of CVD^25^, and the larger CLD is associated with the increased arterial wall stress^26^.

Moreover, Sugawara et al.^26^ reported that the CLD and age are significant independent determinants of the carotid arterial compliance, and they might compensate for the age-related arterial stiffness increase in postmenopausal women. Among the different ELN genotypes, significant arterial stiffness was not observed, but significant correlations were found between the age and the CIMT, the CLD, and the CIMT/CLD ratio, and these might be related to the loss of the orderly arrangement of the elastin fibers^27^. Further, it is possible that these correlations influenced the genetic effects in the postmenopausal and elderly women with the AA genotype.

Further studies are required to review the relationship between the ELN polymorphism and the vascular features with respect to the CLD and the CIMT/CLD ratio in elderly women. It is also necessary to perform studies with larger sample sizes and different genders to increase the statistical power for the genetic polymorphism.

The ELN genotypes show the following distribution: 4.1 % are the AA genotype, 26.2 % are the AG genotype, and 69.7 % are the GG genotype. The CIMT value of the GG genotype is significantly lower than those of the other genotypes (AA and AG); however, no differences were identified regarding the other vascular features such as the CLD and the CIMT–CLD ratio, even though a significant difference is evident between the CIMT and the CLD_min_ of the ELN alleles. These results suggest that it might be possible to use the ELN gene polymorphism as one of the genetic determinants of vascular disease in both pre- and post menopausal women.
